# Reuse of Occluded Veins During Permanent Pacemaker Lead Extraction: A New Indication for Femoral Lead Extraction 

**Published:** 2002-10-01

**Authors:** Andrew D Staniforth, Richard J Schilling

**Affiliations:** 1 Specialist Registrar Cardiology, St Bartholomew's Hospital, London, UK; 2 Consultant Cardiologist, St Bartholomew's Hospital, London, UK

**Keywords:** pacemaker, lead extraction, femoral, drag-through

## Abstract

**Objectives:**

This study examined the utility of a novel technique for reuse of thrombosed veins when extracting permanent pacemaker leads via a femoral vein approach.

**Background:**

Although lead extraction permanent pacemaker using a femoral approach has advantages over the subclavian approach, it cannot be used to provide access for a new lead using currently employed techniques. This is important because up to 23% of patients have occluded veins after permanent pacemaker implantation.

**Methods:**

The pacemaker lead to be extracted was released from the generator and retaining sutures at the implantation site. The lead was then grabbed from below using a needle-eye-snare or basket. The lead was then cut short and a drag through technique performed where a guide wire was pushed into the gap between the insulation and the coil. This guide wire was then drawn into the right atrium as the lead was pulled down from below. This guide wire was then used to introduce a sheath through which a replacement lead could be inserted.

**Results:**

A total of 34 consecutive patients (21 male, aged 63±14 years, mean±SD) had 57 (1.7/patient) leads extracted. Fourteen patients required implantation of a new system and were suitable for immediate lead replacement using the drag through technique. All leads were successfully extracted, with 5 partial successes (9.1% of leads). The drag-through technique was successful in all, including 4 with subclavian vein occlusion. Procedure and fluoroscopy times, including the time required for implantation of a new system, were 143±65 mins and 31±23 mins respectively. There were no complications and hospital stay was 1.6±1.2 days for patients undergoing the drag-through procedure.

**Conclusion:**

The drag-through technique can be successfully used to provide access in order to replace pacemaker leads removed using a femoral approach.

## Condensed Abstract

Thirty-four patients had 57 leads extracted using a femoral approach. In those patients requiring immediate replacement of a pacing lead access for a new lead was achieved in all cases by loosely attaching a guide wire and dragging it into the right atrium behind the explanted lead. A sheath was then introduced over the guide wire and the new lead introduced

## Introduction

Permanent pacemaker lead extraction using a minimally invasive percutaneous approach has been made possible with the development of specialised equipment and techniques [1]. Further developments such a laser assisted lead extraction have facilitated this [2,3] but despite these developments the major complication rate of these procedures stands at approximately 1-3% [2,4]. One of the important reasons for this is that the fragile subclavian vein, superior vena cava and right atrium may tear during the process of cutting away the fibrous tissue that enfolding the leads in these regions. Drawing the body of the lead, which has the narrowest profile (cf. the tip and electrodes), through this fibrous sleeve from below using a femoral approach, avoids the need for this dissection and should prevent these important complications. A further advantage of the femoral approach is that the dissection down to the subclavian vein, required when using a superior approach, can be painful and time consuming. This is not required when using a femoral approach and therefore the procedure can be performed under local anaesthetic. The major disadvantage of the femoral lead extraction approach is that it does not provide access for a replacement lead which can be a problem in the large number of patients with permanent pacemakers who have subclavian vein occlusion [5]. This study investigated the feasibility of a modification to the conventional femoral lead extraction technique that provides venous access allowing the introduction of a new pacing lead along the channel occupied by the extracted lead.

## Methods

### Patients

All patients requiring lead extraction were included in this study. Patients were considered unsuitable for the reintroduction of pacing leads if there was evidence of infection, if they had a functioning system in addition to failed leads or the pacemaker lead had failed because of crushing between the first rib and the clavicle. Otherwise introduction of a new lead using the extracted lead channel was attempted in all patients.

### Lead Extraction Procedure

The patients were taken to the cardiac catheter laboratory in a fasted state having signed informed consent. A temporary pacing wire was positioned at the right ventricular apex, under local anaesthetic, via the left femoral vein. Sedation and analgesia were given in the form of midazolam and diamorphine at the patient's request. The pacemaker implantation site was infused with 1% lignocaine local anaesthetic and opened along the previous insertion scar. The generator was then removed from its pocket and the lead/s requiring extraction were cut near the generator. The lead was freed to the point where it was fixed by retaining sleeves/sutures and these sutures were removed. A 16 French sheath was inserted under local anaesthetic in the right femoral vein and either a basket and deflecting wire (Byrd femoral workstation) or a needle-eye-snare were introduced and the lead snared.

**Figure 1:**
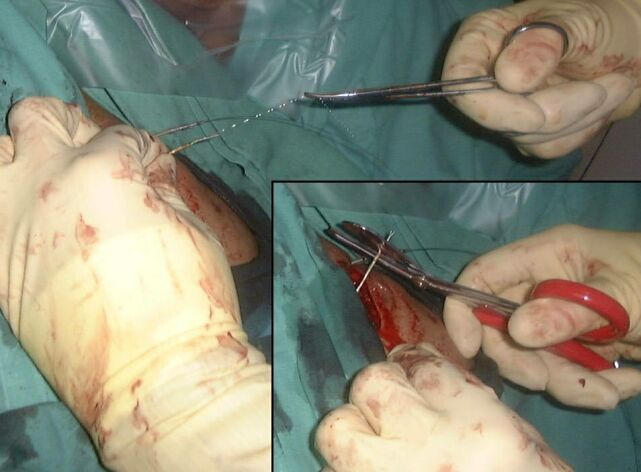
The generator has been removed and the lead to be extracted cut short. Retaining sutures and sleeves have been taken off the lead to be extracted. The coil has been grasped with artery clips and pulled out from the insulation. The coil is then cut at the point where it extends from the insulation (inset), which leaves a gap between coil and insulation. Note that a functioning atrial lead has been left undisturbed (with a stillette inserted).

**Figure 2:**
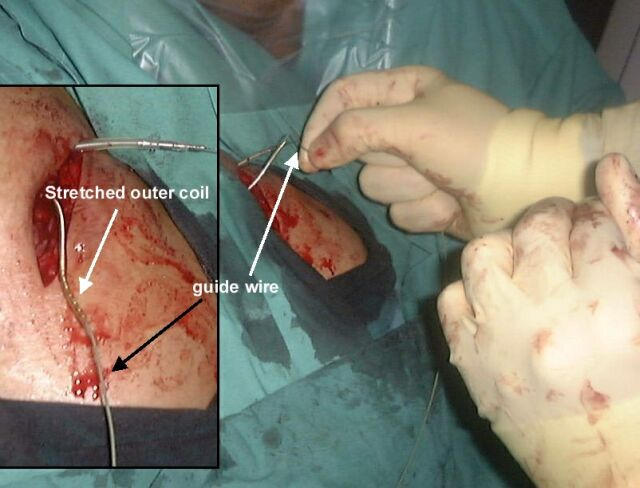
A guide wire is pushed into the gap between the coil and the insulation so that it becomes fairly firmly attached to the lead.

**Figure 3:**
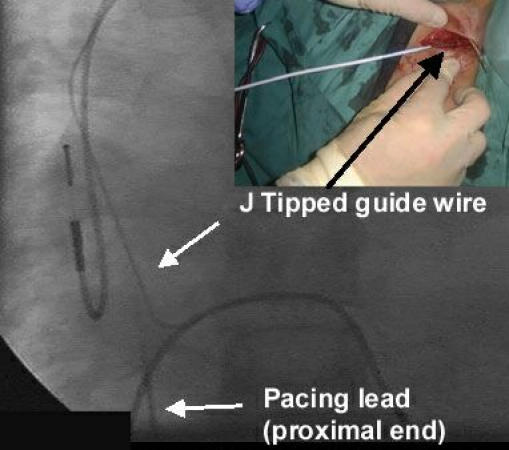
The guide wire has been pulled in behind the lead and has been detached at the level of the right atrium. Note that the atrial lead has been left undisturbed and can be used for the new pacing system. Inset: the guide wire is used to pass a "peel-away" sheath to allow introduction of a new pacing lead.

 The drag-through technique was then performed in the following way. The pacing lead was cut short with a one inch of lead left exposed. The outermost electrode coils of the pacemaker lead were then grabbed with an artery clip and stretched out from the lead insulation (Figure 1). While tension was applied to the coil it was then cut at the outer insulation, resulting in the coil inside the lead being partially unwrapped, leaving a gap between the outer insulation and the coil. A 140cm 0.35" J-tipped guide wire was then introduced into the gap left between the coil and the insulation and pushed in until it was firmly attached (Figure 2). No other method was used to affix the guide wire and specifically no sutures or ties were used to fix the two together. The proximal end of the pacemaker lead was then pulled down into the right atrium from below while the guide wire was gently fed in, as it was drawn in behind the pacing lead. When the proximal pacing lead/guide wire join reached the level of the right atrium the guide wire was fixed and the pacing lead pulled so that the two separated (Figure 3).

The pacemaker lead was then extracted using previously described conventional counter-traction techniques [6]. The guide wire was then used to introduce a "peel-away" sheath through which a new pacing lead was then passed.

### Definitions

Outcomes of the lead extraction were based on previously published recommendations [7]. Complete success was defined as removal of all material from the vascular space. Partial success was defined as removal of all but a small portion of the lead. Clinical success was defined as achievement of all clinical goals associated with the indication for lead removal.

## Statistics

 Continuous data is presented as means and standard deviations

## Results

### Patients

A total of 34 consecutive patients (21 male, aged 63±14 years, mean±SD) underwent extraction of 57 leads which had been in situ for 7.8±4.4 years (7±3.3 years for drag-through patients). Seventeen patients had infected systems and were therefore not suitable for immediate replacement (Table 1). Of the remaining patients, one required radiotherapy for breast carcinoma on the ipsilateral side, and another had a long section an active fixation lead floating free in the right atrium following a failed attempt to extract it from the superior approach at another centre. Therefore, 15 patients were suitable for lead extraction and immediate replacement of their pacing system, 2 of whom required extraction of an old pacing system in order to implant a defibrillator. The indications for lead extraction in the remaining patients were lead failure in a young patient (<60 years) (n=7), Telectronics accufix lead removal was requested by two patients. One patient requested lead removal for pain and discomfort, whilst in another X-ray identified the cause of lead failure as a crush fracture between the clavicle and first rib such that new leads were implanted via the cephalic vein to prevent this occurring again.

### Procedure

 Leads were extracted using a Byrd femoral workstation in 16 patients (Cook UK Ltd) and a needle eye snare in 17 patients (Cook UK Ltd) and both in 1 patient (table 1). Clinical success was achieved in all cases. Radiographic success was achieved in 100% of leads with 91.2% (52/57leads) complete success; partial success resulting from retention of either the electrode (n=3) or <2cm of the distal end of the lead (n=2). The drag though technique was attempted in 93% (14/15) of patients suitable for immediate replacement of a pacing system on the ipsilateral side. Four of these patients (31%) had total subclavian vein occlusions identified on venography prior to extraction. Achieving venous access via the drag-through technique was successful in all patients and was used to extract and replace 19 pacemaker leads. Four lead extraction procedures were performed under general anaesthetic (11.7% of subjects). The indications for general anaesthetic were concurrent defibrillator (ICD) implantation (n=2), septicaemic shock resulting in confusion (n=1) and a heavily infected wound requiring extensive debridement (n=1). Two patients undergoing a drag-through procedure had 3 leads removed including 2 previously abandoned ventricular leads. Eight patients had one lead extracted only (5 ventricular, 2 ICD, 1 atrial) with a functioning pre-existing lead left undisturbed in 3 cases (all atrial). A single guide wire was dragged-through and used to introduce 2 sheaths allowing upgrade from a single chamber to a dual chamber pacemaker in 1 patient, otherwise individual guide wires were introduced for each lead being replaced. Mean procedure and fluoroscopy times for all femoral lead extractions, including the time required for implantation of a new system, were 143±65 minutes and 31±23 minutes. For the patients undergoing a pacemaker lead drag-through and re-implantation procedure the times were 156±71 minutes and 41±28 minutes compared with 134±62 minutes and 25±18 minutes for those not undergoing drag through (p=NS). Mean stay in hospital was 4.5±6.7 days for all patients(presumably should quote non-drag sub-group rather than total group) and 1.5±1.1 days for the drag-through patients, reflecting the lack of infection in these patients.

## Discussion

 Permanent pacemaker lead extraction has published major complication and mortality rates of 1.4% and 0.04% [4] and the results are similar using laser assisted lead extraction with major complication and mortality rates of 3.2% and 0.6% [2]. Perforation of the superior vena cava (SVC) or the lateral right atrium contributes to complications observed during extraction from a superior approach. Extracting pacemaker leads from a femoral approach should avoid these problems because there is no dissection performed in the right atrium or superior vena cava. Most published series of femoral lead extraction have described cases that have already had an attempt at extraction from a superior approach, but a recently published series in which femoral lead extraction was the primary approach in most patients have reported no major complications or deaths in 78 patients, supporting the theoretical safety of this approach [3].

 The limitation of femoral lead extraction is that there is no access for replacement of a new pacing system. This is of particular relevance in those patients with subclavian or brachiocephalic vein occlusions, which occurs in up to 23% of patients with permanent pacemakers [9]. Lead extraction and replacement of the lead on the same side avoids the possibility of bilateral subclavian occlusions, failure to cross a superior vena or the need to implant a femoral system. Recent reports have demonstrated that replacing pacemaker leads through occluded veins is possible particularly when using a laser-assisted approach [10]. This study has demonstrated that replacement of permanent pacing leads is possible using the cheaper femoral approach even when the veins are occluded. This was achieved without complication and was performed under local anaesthetic in most cases. Obliteration or occlusion of all useable veins is currently a class I indication for lead extraction as recommended by the North American Society for Pacing and Electrophysiology (NASPE) [7]. but occlusion of the ipsilateral subclavian vein is not included as an indication. If, as limited data published so far has suggested, the femoral approach is safer than a superior approach, the indications for lead extraction could be extended to include ipsilateral subclavian venous occlusion as a class 2 indication based on the technique described in this study.

## Conclusion

Replacement of non-functioning pacemaker leads using the same route of access as the extracted lead can be safely achieved using a femoral approach. In the majority of cases the femoral lead extraction can be used as the approach of choice. Occlusion of the subclavian vein is a new indication for lead extraction using a femoral approach.

## Figures and Tables

**Table 1:**
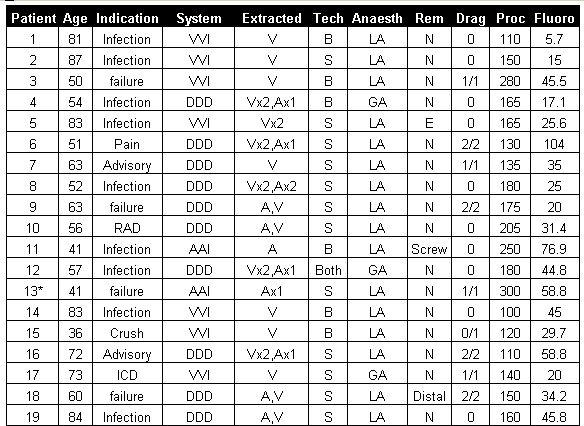

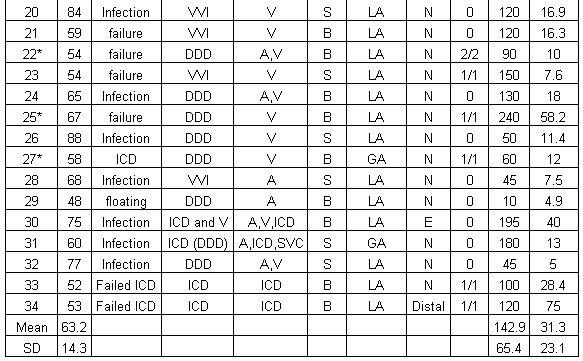


* indicates patients with venous occlusion of the ipsilateral veins. *Indication*: **Pain**=painful pacemaker; **Failure**=lead failure and the patient is young (> 60)); **Advisory**=an advisory lead and the patient has requested lead extraction; **ICD**=patient required upgrading of pacemaker to ICD or replacement of a failed defibrillator lead. *System*: the system in situ requiring extraction; **DDD**=dual chamber; **VVI and AAI**=single chamber. *Extracted*: the number of each type of leads extracted. *Tech*: the system used to perform lead extraction; **S**=needle eye snare; **B**=Byrd femoral workstation. *Anaesth*: **LA**=local; **GA**=general anaesthetic. *Rem*: indicates whether any lead fragments were retained;  **N**=none; **distal**=<2cm of distal lead was retained. *Drag*: how many leads were replaced by the drag through technique; */*=number of leads dragged through/number of requiring extraction. *Proc and Fluoro*: procedure and fluoroscopy time (mins) including time for new implants.
